# FDCNet: Presentation of the Fuzzy CNN and Fractal Feature Extraction for Detection and Classification of Tumors

**DOI:** 10.1155/2022/7543429

**Published:** 2022-05-06

**Authors:** Sepideh Molaei, Niloofar Ghorbani, Fatemeh Dashtiahangar, Mohammad Peivandi, Yaghoub Pourasad, Mona Esmaeili

**Affiliations:** ^1^Faculty of Electrical and Computer Engineering, Tarbiat Modares University, Tehran, Iran; ^2^High Point University, Department of Mathematical Sciences, High Point, NC, USA; ^3^Department of Electrical Engineering, Golestan University, Gorgan, Iran; ^4^Fachbereich Wirtschafts- und Rechtswissenschaften, HTW Berlin - University of Applied Sciences, Berlin, Germany; ^5^Department of Electrical Engineering, Urmia University of Technology, Urmia, Iran; ^6^Department of Electrical and Computer Engineering, University of NM, Albuquerque, NM 8731, USA

## Abstract

The detection of brain tumors using magnetic resonance imaging is currently one of the biggest challenges in artificial intelligence and medical engineering. It is important to identify these brain tumors as early as possible, as they can grow to death. Brain tumors can be classified as benign or malignant. Creating an intelligent medical diagnosis system for the diagnosis of brain tumors from MRI imaging is an integral part of medical engineering as it helps doctors detect brain tumors early and oversee treatment throughout recovery. In this study, a comprehensive approach to diagnosing benign and malignant brain tumors is proposed. The proposed method consists of four parts: image enhancement to reduce noise and unify image size, contrast, and brightness, image segmentation based on morphological operators, feature extraction operations including size reduction and selection of features based on the fractal model, and eventually, feature improvement according to segmentation and selection of optimal class with a fuzzy deep convolutional neural network. The BraTS data set is used as magnetic resonance imaging data in experimental results. A series of evaluation criteria is also compared with previous methods, where the accuracy of the proposed method is 98.68%, which has significant results.

## 1. Introduction

The soft and spongy mass of tissue protected by the cranial bone is called the brain, which has three thin layers of tissue (meninges). A watery fluid circulates in the spaces inside the brain (Cerebral Spinal Fluid). The brain controls both voluntary (such as walking or speaking) and automatic activities (such as breathing). The brain is responsible for memory, emotions, and personality in addition to the five senses (sight, hearing, touch, taste, and smell). An intricate network of nerves connects the brain and body. A benign brain tumor does not contain cancer cells, but a malignant brain tumor does. It is vital in the health system to identify and diagnose brain tumors. Early detection of cancer can help a person live longer. The brain has a variety of tissues that result from the accumulation and overgrowth of cells to cause brain tumors. Some of the symptoms of brain tumors are headaches, seizures, mood swings, and movement disorders. There are three treatments for brain tumors: surgery, radiation therapy, and chemotherapy. Each symptom has side effects that have been studied in [1].

MRI images are widely used due to their excellent spatial resolution and tissue contrast. The extent to which a tumor threatens depends on different factors such as the type, the location and size of the tumor, and how it spreads and develops. MRI elements must be divided into four categories to diagnose: white matter (WM), gray matter (GM), Cerebral Spinal Fluid (CSF), and abnormal brain tissue (tumor). The purpose of brain tumor segmentation is to isolate various tumor tissues such as active cells, dead tissue center, swelling in normal white matter (WM), gray matter (GM), and Cerebral Spinal Fluid (CSF). Due to the high medical diagnosing capabilities of computer science, such as image processing and machine vision, these have been used extensively in various fields of science. This study provides a comprehensive approach to the diagnosis and classification of benign and malignant brain tumors. Studies and innovations of the proposed method are included in the following:

1. A fuzzy deep convolutional neural network method called FDCNet is proposed for diagnosing and classifying brain tumors using MRI images. The FDCNet approach integrates fuzzy theory with the remaining network, generating fuzzy rules using an adaptive learning algorithm. It examines fuzzy and deep indexes to construct the features of brain tumors and also solve the problem of uncertainty.
2. A deep fuzzy convolutional network model is designed to detect and classify brain tumors from MRI images using the representation of tensor data to examine the temporal and locative features of brain tumors. FDCNet structure is optimized by exploring the number of layers in the regression model and functions. The FDCNet method has high identification, detection, and classification capabilities.
3. The proposed approach in the BraTS data set is trained and tested to compare and observe the assurance that the proposed method can measure in terms of evaluation criteria. The main advantage of MRI imaging detection and classification systems on brain tumors is the improvement of evaluation criteria in the same conditions of the data set with the presented methods that can be used to determine the efficiency of the methods.

In this paper, the following sections are presented: (1) “Introduction” section describes the problem, details the contribution, and shows the novelty of the approach. (2) “Literature Review” section provides information on recent research investigating the problem and the methodology. (3) “Methods and Materials” section provides an overview of the approach characteristics and the strategies proposed. (4) “Results and Discussion” section aims to analyze the prediction outcome using the provided technique. (5) “Conclusion” section concludes the presentation by providing an overview of the overall results and future directions.

## 2. Literature Review

Various studies have been conducted on brain tumors diagnosis. The fragmentation has been discussed as a deep learning technique to detect brain tumors from MRI images using the convolutional neural network in [2]. The BraTS data set has been considered as the input data. This study indicates an improvement in fragmentation to classify and detect brain tumors from data sets with relatively high accuracy. Another study [3] discussed fragmentation operations to identify and classify brain tumors from MRI images using a generative adversarial network (GAN). This method combines the production model with the discriminatory model and uses the generative adversarial network instead of a conditional random field as a high-level smoothing method. The proposed method was trained and tested in the BraTS data set. It can be demonstrated that the proposed method gains a competitive result and the usage of a generative adversarial network improves the network's performance.

Furthermore, this approach only takes about 10.8 seconds to perform high-precision segmentation and brain tumors diagnosis compared to other preconvolutional neural network-based methods. The average accuracy of this method has been 94.5%. Similarly, a generative adversarial network (GAN) in [4] has also been used for the segmentation, detection, and classification operation of brain tumors from MRI images. The optimization operation of the generative adversarial neural network has been performed using a method called progressively growing GAN named PGGAN. The main features of this method were as follows: considering the size of the images as 256 × 256 pixels in an integrated form, also illuminance intensity, and the position of the masses and edges, where the average accuracy of this method was 95%. Using a convolutional neural network with a combinational method called neutrosophic expert maximum fuzzy sure entropy has been studied to detect and classify brain tumors from MRI images in [5]. Liu et al. (2021) suggested a new method to produce super-resolution (SR) ultrasound images that maintains perceptual consistency. The PSNR/IFC/SSIM, inference efficiency, and visual impacts evaluations using the benchmark data sets CCA-US and CCA-US demonstrate that our proposed methodology is both effective and superior to the existing state-of-the-art approaches [6]. According to Zhang et al. (2020), a new method is proposed for optimizing clinical pathway queries in e-healthcare systems while preserving privacy [7]. Tang et al. (2018) described a technique for identifying the origin of tumors using tissue-specific miRNA and DNA methylation indicators [8]. In the general approach called NS-CNN-EMFSE, the final classification uses a support vector machine and *K*-Nearest Neighbor method. K-fold has also been used for cross-validation and accuracy for evaluation. BraTS data sets are considered input data, and the results show that the classification accuracy is 96.52%. Also, deep neural network convolution is used by amplifying extensive data in the diagnosis of brain tumors from MRI images and BraTS data sets in [9]. Extensive data amplification has been considered to improve diagnostic and classification operations. The accuracy of the diagnosis and classification results was 95.5%. Liu et al. (2021) proposed a method for style and character inpainting. Based on the CGAN network repair style, the paper proposes font content that is similar to the correct content [10]. Chen et al. (2021) suggested using LSTM and clustering algorithms to predict human N6-methyladenosine (m6A) sites [11]. Ahmadi et al. (2022) presented a novel model for energy forecasting that incorporates a deep convolutional neural network with fuzzy wavelets and a PSO method [12]. An expert system in [13] is considered for detecting brain tumors from MRI images. The research approach is to use the fuzzy *C*-means method or FCM in fragmentation operations and data training in the convolutional neural network. The membership functions (MFs) in fragmentation have been an effective and well-performing feature identification method. Also, another method named Extreme Learning Machine (ELM) has been used in the final classification section. The approach which uses BraTS data images as super-pixel is called SR-FCM-CNN-ELM. Its accuracy rate is 98.33%. In [14], Discrete Wavelet Transform (DWT) has been used during the training and testing of brain MRI data to classify and segment tumors in repetitive rings of the convolution neural network. Also, an innovative noise reduction method called Partial Differential Diffusion Filter (PDDF) has been proposed in this study, reducing salt and pepper noise to a large extent in the BraTS data set. The accuracy of this method to detect and classify brain tumors from MRI images in the BraTS data set is 98%. Xu et al. (2021) suggested a technique for segmenting and detecting tongue images using a deep convolutional neural network called the Multitask Joint Learning Model [15]. In [16], brain tumor detection in MRI images and also image segmentation operations using the Genetic Algorithm (GA) are presented, and the Discrete Wavelet Transform (DWT) has been used for image improvement. In another study in [17], Ant Colony Optimization (ACO) and the Genetic Algorithm (GA) have improved the implementation of MRI image fragmentation. But the general basis of brain tumor detection methods is derived from machine image processing and vision systems. Magnetic Resonance Spectroscopy (MRS) and Diffusion-Weighted Imaging (DWI) in the supratentorial area of brain tumors are among the studies conducted in this field of image processing and computer vision methods [18]. Ahmadi et al. (2021) described a unique hybrid strategy for user selection in Federated Learning using Deep-Q-Reinforcement Learning and Spectral Clustering. On the three data sets, MNIST, Fashion MNIST, and CIFAR-10, the achieved reductions in communication rounds are 51%, 25%, and 44%, respectively [19]. Eslami et al. [20] have investigated an attention-based multi-scale convolutional neural network (A+CNN) for multiclass classification in road images. Ala et al. [21] examined a variety of hypotheses to meet the analysis and different factors related to hospital patients as well as calculating WOA and NSGA. Abadi et al. [22] used a hybrid salp swarm algorithm and genetic algorithm for identifying and scheduling COVID-19 patients for care. It was shown by Mahmoudi et al. [23] that the adjusted SEQIER model is a good fit to the real COVID-induced daily death data, as it could capture the nonlinearities of the data well. Sadeghipour et al. [24] used the firefly algorithm and intelligent systems to detect breast cancer. According to Ahmadi et al. [25], they used convolutional neural networks (CNNs) to segment tumors in seven types of brain diseases: Glioma, Meningioma, Alzheimer's, Alzheimer's plus, Pick, Sarcoma, and Huntington disease. Yazdani et al. [26] proposed in this paper an improved version of extended classification system “trained” using examples from existing data. This would help to identify and avoid attempts to interfere with computer systems during application phases. Rezaei et al. [27] proposed a method for segmenting hand parts using depth maps without any additional effort involved in obtaining segmentation labels. Mobasheri et al. [28] reviewed the immunological findings in COVID-19 and the current reports on autoimmune diseases associated with this disease. In their study, Hastantabar et al. [29] presented two algorithms, including a deep neural network (DNN) and a convolutional neural network (CNN) directly using images of the lungs. Dorosti et al. [30] presented a general model to identify the correlation of different parameters in a GC tumor place and tumor size. The medical documents of GC patients consist of the dataset of this study. Yan et al. (2020) developed a method of determining whether waxy or regular maize starches are more digestible after thermal treatment using plasma-activated water [31]. Shi et al. (2021) investigated the effects of ultrasonic intensity on wheat starch and monoglyceride combinations as well as its influence on the quality of Chinese steamed bread [32]. Yang et al. (2019) examined methods for improving the quality of HEVC-compressed videos. Finally, they created a prototype that demonstrates our TQEO concept in action [33]. The study deals with the tumors in the cerebellum region approach. Diffusion-Weighted Imaging is an imaging technique that uses the Brownian motions of water molecules to generate contrast in MRI images. Another method using edge detection is presented in [34], which has modeled the upgraded method of edge detection for segmenting the brain tumor in MRI images called the Sobel edge detection method. The steps of the segmentation algorithm have four stages: (a) finding the image gradient with the Sobel edge detector operator, (b) calculating the dependent threshold image repetition in the cycle, (c) applying the closed contour algorithm, and (d) fragmentation of objects in the image based on the pixel intensity between the closed contours. It is remarkable to use combinational simpler methods in machine vision and image processing. MRI images of brain tumors were performed by watershed segmentation and the Hierarchical Clustering Algorithm [35]. The hierarchical clustering methods used in this study include the *K*-Nearest Algorithm, the *K*-Farthest Algorithm, and the Minimal Spanning Tree method. Also, in [36], anisotropic diffusion based on fragmentation and the pattern based on group classification of MRI images to detect a brain tumor based on a support vector machine and fragmentation with FCM were performed. According to Xu et al. (2019), omnidirectional videos were assessed for visual quality. Researchers in this study demonstrate that both subjective and objective methods of video quality assessment for omnidirectional videos increase state of the art [37]. In their study, Sheng et al. (2021) examined near-online monitoring in blockchain-based edge devices with constraints on cooccurrence [38]. Neural networks have been utilized in various fields for detecting brain tumors. In [39], an unsupervised learning method by a clustering technique for identifying a brain tumor in MRI images based on fragmentation has been proposed. Using the self-organizing mapping (SOM) neural network (NN) method with fuzzy *K*-means (FKM) algorithm has been the main proposed method of this research. In a similar study in [40], MRI imaging was performed to detect a brain tumor based on a neural network of self-organizing mapping and entropy-gradient clustering. The study by Chen et al. (2021) examined cellular autophagy and noncoding RNA's role in colorectal cancer [41]. He et al. (2020) explored the use of multibranch deep residual learning for clustering and beamforming in user-centric networks [42]. In a different approach in [43], MRI images' brain tumor detection and fragmentation operations have been performed using two techniques. The first one uses the Gabor filter method and Wavelet Transform. In contrast to that, statistical features methods were used, such as Linear Support Vector Machine (LSVM), Radial Basis Function (RBF), *K*-Nearest Neighbor (KNN), Euclidean *K*-means and blocked *K*-means, and Sparse Representation. Combining neural networks with other methods is also common in tumor diagnosis [44], which provides the performance analysis of a classification method for identifying and detecting brain tumors from MRI images based on the neural-fuzzy network [45]. Niu et al. (2021) used ensembles of convolutional neural networks to detect sgRNA on-target activity in four crops. These findings have implications for agricultural gene editing and academic research [46]. Sun et al. (2021) evaluated a babysitting package for the analysis of retrospective and freshly produced RNA-seq data using both alignment-based and alignment-free quantification methods [47]. Zhang et al. (2020) examined the privacy-preserving clinical pathway query optimization for healthcare networks [7]. Olowookere [48] created two- and three-class models for breast cancer detection and classification using a deep convolutional neural network and fuzzy support vector machines. According to this study's addition to knowledge, the hybridization of deep convolutional neural networks with fuzzy support vector machines improved the identification of malignant and noncancerous breast cancers in both binary and three-class classification situations. Ragab et al. [49] created a novel ensemble deep-learning-enabled clinical decision support system that uses ultrasound pictures to diagnose and classify breast cancer. The researchers devised an optimum multilevel thresholding-based picture segmentation approach to detect the tumor-affected areas. The researchers also created a feature extraction ensemble comprising three deep learning models and an effective machine learning classifier for breast cancer diagnosis. The research provides a method for radiologists and healthcare practitioners to aid in the categorization of breast cancer [49]. Liu et al. (2021) investigated perception constancy ultrasound image super-resolution using self-supervised CycleGAN. Using the benchmark data sets CCA-US and CCA-US, we evaluated PSNR/IFC/SSIM, inference efficiency, and visual impact of our proposed approach for comparison with existing state-of-the-art approaches [6]. Tang et al. (2018) examined the possibility of detecting tumor origins using tissue-specific miRNA and DNA methylation indicators. They created a user-friendly website that enables users to determine the origin of tumors by uploading miRNA or DNAm profiles relevant to their research [8]. Sharifi et al. (2021) used convolutional neural networks to investigate the experimental and numerical diagnosis of tiredness foot. The current CNN technique outperforms existing methods and may be employed in the development of future fatigue detection systems [50]. Ahmadi et al. (2021) developed a novel classifier for brain tumor identification based on fuzzy logic and wavelet-based neural networks [51]. Liu et al. (2021) concentrated on issues related to font inpainting. This paper will discuss how to restore broken fonts depending on their style in order to do it more effectively [10]. Rao and Karunakara [52] concentrated on effective segmentation and classification using machine learning models to diagnose tumor development and therapy procedures. Preprocessing, segmentation, extraction, selection, and classification are phases in the suggested approach for efficiently detecting brain tumors. Higher detection accuracy allows for fast and accurate diagnosis, which may save people's lives. As a result, this tumor detection and classification results indicate enhanced performance compared to baseline models. Xu et al. (2020) classified and segmented tongue pictures using a deep neural network. The findings indicate that their technique is extremely compatible with human perception [15].

Maniraj and Maran [53] suggested a hybrid deep learning strategy based on 3D wavelet subband fusion. It is a noninvasive, objective way to examine skin photographs. The provided approach's performance findings on PH2 database photos show that it can efficiently classify normal, benign, and malignant skin images with 99.33 percent average accuracy and more than 90% sensitivity and specificity.

## 3. Methods and Materials

### 3.1. An Overview of Deep Convolutional Neural Networks

Deep learning involves using multiple levels and layers of learning to model high-level abstract concepts using machine learning, artificial intelligence, and other technologies. The idea of deep learning, which was inspired by the natural structure of the human brain and with the help of new facilities and technologies, has been able to achieve considerable success in many areas related to artificial intelligence and machine learning. Recently, artificial intelligence problems can be solved with deep learning algorithms. The most important benefits of deep learning are as follows:

- Automatic features learning
- Multilayered features learning
- High accuracy in results
- High generalization ability and identification of new data
- The potential to create more capabilities and applications in the future
- Identifying the data features regardless of the location

Deep neural networks, such as convolutional neural networks (CNNs) and ConvNets, are often used for visual and audio processing. Convolutional neural networks use multilayered perceptron types to minimize preprocessing. These are sometimes referred to as shift invariant or space invariant neural networks. Its structure is inspired by the biological processes of the cat's visual cortex such that single neurons only respond to stimulation in a limited area, which is called the reception area. The reception areas of the various neurons overlap in inconsiderable parts to cover the entire field of view. Compared to other data classification approaches, convolutional neural networks use fewer data preprocessing, meaning that the network meets the standard learned manually in previous approaches. This essential advantage is independence from last knowledge and human manipulation in convolutional neural networks. Numerous applications of neural networks have been proposed, including computer vision, suggestive systems, and natural language processing. One of the most significant deep learning techniques is convolutional neural networks, which effectively train many layers. In many applications, this strategy is very common and effective. In general, convolutional neural networks consist of three layers: convolution, pooling, and fully connected layers. Each layer has a distinct function. Feed-forward and backpropagation training are the two stages of convolutional neural networks.

In the first step, the input is provided to the network, and this operation amounts to nothing more than a dot product between the input and the parameters of each neuron. After this, each layer is convolutionalized. The network output is then calculated to determine the network error rate, which is used to adjust the network parameters or, to put it differently, the network training itself. Outputs are compared with a loss function. The backpropagation step is then initiated based on the calculated error rate. In this step, the gradient of each parameter is computed using the chain rules, and each parameter is adjusted according to its influence on the network error. After the settings have been adjusted, the subsequent feeds begin in reverse order. The network's training is complete once a sufficient number of these processes have been completed.

The proposed approach has three main phases, including preprocessing operations for image enhancement, features segmentation and extraction, and a final classification for determining the type of tumor. Features segmentation and extraction operation based on the fractal model are presented in a deep neural network of fuzzy convolution phases and training section, and afterward, the classification occurs. At first, the brain MRI input images must be normalized. Brain MRI images also have a series of noises. Therefore, an initial optimization must be made on these images so that the process occurs in the main sections to achieve a significant result. In the preprocessing phase, input data, which are tumorous and nontumorous images, are normalized and enhanced to increase the efficiency of the system's recognition if necessary.

### 3.2. Image Optimization and Preprocessing Operations

Image optimization and preprocessing operations are performed in the following steps:

**Step 1 .**

Normalize the image size for changing the image size to a default size such as 256 × 256. Brain tumor segmentation systems use 128 × 128 or 256 × 256 pixels or the normal collection size based on a unit. The other issue in normalization is called camera distance from the tumor. According to equation (1), all of the measured values relative to this quantity are normalized by an amount named BASE.

(1) $$\begin{array}{c} \text{BASE}=\sqrt{{\left(x_{5}-x_{10}\right)}^{2}+{\left(y_{5}-y_{10}\right)}^{2}.} \end{array}$$

The tumor is considered the reference point to determine the focal length in different images that should be measured relative to a fixed reference, which is assumed to be the origin of face focal length compassion. The coordinate's origin is obtained from the following equations:

(2) $$\begin{array}{c} x_{0}=\frac{x_{5}-x_{10}}{2}, \end{array}$$

(3) $$\begin{array}{c} y_{0}=\frac{y_{5}-y_{10}}{2}. \end{array}$$

**Step 2 .**

Conduct histogram integration of the images on images that are dark and should be bright enough to extract important features of the brain tumor, including the intended tumor tissue.

**Step 3 .**

Intermediate filtering eliminates possible noises.

**Step 4 .**

High-pass filtering is the process of traits extraction based on brain tumor generalities. It is possible to get better results from edge finding and fragmentation methods. High-pass filtering emphasizes details such as edges, which thereupon increases edge detection efficiency.

**Step 5 .**

Eliminate the rotational effect obtained from the following equation and also conducted to adjust the image level.

(4) $$\begin{array}{c} \theta =\arctan\frac{y_{5}-y_{10}}{x_{5}-x_{10}}. \end{array}$$

In this relation, *θ* is the BASE angle relative to the horizon for eliminating the tumor rotation. The action gives rise to calculating the new coordinates relative to the origin obtained from

(5) $$\begin{array}{c} y_{a}=y_{b}-y_{\text{original}}. \end{array}$$

### 3.3. Features Segmentation and Extraction Operation

The system designer has no control over the operating environment in applications, such as automatic targeting. The usual approach is to focus on selecting the type of sensor that is most likely to increase the desired targets and reduce the share of irrelevant visual details. Most image segmentation algorithms are based on one of the two basic features of brightness intensity, discontinuity, and similarity. Discontinuity methods divide an image like edges based on sudden changes in brightness intensity. Simulation methods are based on dividing an image into areas similar to each other according to predefined criteria. Thresholding, region growing, cutting, and merging of areas are examples. This study will use fractional-based morphological descriptive-based segmentation descriptors. The morphological operation refers to a branch of biology dealing with shape and structure. The term “mathematical morphology” is used to extract image components useful in expressing features and describing the shape of areas such as boundary areas, frameworks, and convex hulls. The language of mathematical morphology is set theory and a unified and powerful way to deal with image processing issues. In mathematical-based morphology, sets are represented as objects in an image. The fractal model is used for segmentation and extraction operations, which works based on texture, brightness intensity, and edge features. Initially, the durability section identifier is used. For *A* and *V* sets in *Z*, durable areas of *A* and *B* are displayed as *A*⊖*B*, defined as

(6) $$\begin{array}{c} A⊖B=\left\{z|{\left(B\right)}_{z}\subseteq A\right\}. \end{array}$$

It indicates that the erosion of *A* by *B* is the set of all points *z* such that *B* is contained in *A* that the components do not share between *B* and the background, and the durability regions can be represented as

(7) $$\begin{array}{c} A⊖B=\left\{z|{\left(B\right)}_{z}\cap A^{c}=\emptyset \right\}. \end{array}$$

According to (7), *A*^*c*^ is the complement of set *A* and ∅ is the null set. Then, the self-similar operator is used. If *A* and *B* are set in *Z*^2^, the dilation of *A* by *B*, denoted *A⨁ B*, will be displayed in the form

(8) $$\begin{array}{c} A⊕B=\left\{z{\left(\hat{B}\right)}_{z}\cap A\neq \emptyset \right\}. \end{array}$$

In (8), *A* ⊕ *B* is the reflection of *B* around its origin and shifting this reflection by *z*. Therefore, the dilation of *A* by *B* is the set of all *z* displacements. According to the interpretation, the expanded relationship will be in the form

(9) $$\begin{array}{c} A⊕B=\left\{z|\left[{\left(\hat{B}\right)}_{z}\cap A\right]\subseteq A\right\}. \end{array}$$

According to (9), *B* is a structural component, and *A* is a set of image objects on which self-similarity should be conducted, used for the segmentation of boundary extraction in the fractal model. The boundary of a set *A* shown with *β*(A) can be found first with the durable operator A with B and then subtracted between set *A* and its durability, which is in the form

(10) $$\begin{array}{c} \beta \left(A\right)=A-\left(A⊙B\right). \end{array}$$

According to (10), *B* is a suitable structural component. After the image segmentation is done, the feature extraction step will be performed. Brain tumor features extraction from MRI images is a vital process that significantly impacts classification results. The proposed method is obtained by extracting the necessary information from the data. Therefore, only intrinsic features are selected for data classification. Also, the fractal model has been used to extract the feature. The feature selection has been used in order to deal with high input features and also to reduce the dimensions and identify the most relevant features causing the sufficient separation of different classes. The feature selection method is a sensitive one; thus, insufficient features reduce the classification efficiency, while a larger set of features does not always give better accurate identification results.

Using covariance analysis, fractal features from the image were extracted, generating eigenvalues and minimizing the dimensions. The input images for the fractal algorithm must be of the same size. The two-dimensional matrix of a single image is referred to as a single vector. The first step in the fractal algorithm is to upload training images. These should be grayscale images with a certain resolution. An image containing both the background and the tumor will not be able to provide an accurate diagnosis of the tumor. Each image is converted to a column vector by adding lines, and the images are loaded from a matrix of size *M* × *N*, where *N* is the number of pixels and *M* is the number of images. It is necessary to compute the average image to obtain the standard deviation for each original image. We then construct the covariance matrix and obtain the eigenvalues and eigenvectors of the covariance matrix that correspond to the tumor's values. Light intensity, edges, and texture are among the primary features of this research. The number of training images *M*, the average of the images *F*_*i*_, and the number of images *L*_*i*_ denote the number of images in the vector *T*_*i*_. Initially, there are *M* images, each of which contains the *N* × *N* dimension. Equation (12) describes the process of averaging an image in a three-dimensional *N* space.

(11) $$\begin{array}{c} A=N\times N\times M, \end{array}$$

(12) $$\begin{array}{c} Fi=\frac{1}{M}\sum_{t=1}^{m}Tt. \end{array}$$

Finding the standard deviation is an important issue in the fractal algorithm, which is also calculated through equation (13) and the covariance matrix from (14).

(13) $$\begin{array}{c} \text{Variance}=\frac{1}{M}\sum_{t=1}^{m}Tt, \end{array}$$

(14) $$\begin{array}{c} \text{Cov}=AA^{T}. \end{array}$$

*A*=⌊Variance_1_, Variance_2_,…, Variance_*n*_⌋ and Cov=*N*^2^*∗N*^2^ are a matrix because *A*=*N*^2^*∗M* is a matrix. So Cov is huge. Eigenvalues are now obtained from Cov using

(15) $$\begin{array}{c} U_{i}=AV_{i}. \end{array}$$

The last step is to choose eigenvectors. A collection of intrinsic state function attributes in the form of *N*(*N*=213) samples in a D-dimensional space {*x*_1_, *x*_2_,…, *x*_*N*_} is provided. *x*_*i*_=*R*^*d*^ and belongs to the C (*C* = 7) class from {*L*_*i*_*i*=1,2,…, *C*}. The fractal algorithm aims to find a linear transmission that maps the original D-dimensional space to the F-dimensional space denoted by *f* < *d*. At *y*_*i*_=*R*^*F*, the new feature vector is situated. Total scatter or covariance matrices are used to describe scattered matrices in the class. They are computed using (16) and (17).

(16) $$\begin{array}{c} S_{T}=\sum_{k=1}^{N}\left(x_{k}-\mu \right){\left(x_{k}-\mu \right)}^{T}, \end{array}$$

(17) $$\begin{array}{c} W_{\text{Fractal}}=\arg\;\max\left[W^{T}S_{T}W\right]=\left[w_{1}w_{2}\ldots w_{f}\right]. \end{array}$$

*μ* is the samples mean and {*w*_*i*_*|i*=1,2,…, *f*} is a set of special *S*_*T*_*F*-dimensional vectors that are associated with the largest *f* eigenvalues. Samples in the new space are *y*=*W*^*T*^*x*, which is *W*_Fractal_ ∈ *R*^fxd^(170*xd*). The main components of the tumor (*s*) are calculated in the training set. Identification operations are formed by tumor designs components in the image space. A comparison is made based on the Euclidean distance of eigenvectors from the main components of the image. The tumor in the image can be identified if the distance is small. On the other hand, if the distance is so large, the image is considered one of the accessories for an independent sample that the system has trained. As a starting point, the training images are read in *N × N* dimensions and converted to *N*^2^ × 1 dimension.

### 3.4. Training and Testing with Deep Fuzzy Convolutional Neural Network Algorithm

An *N*^2^ × *M* training set is also built-in, and *M* is the number of image samples. The average image set is calculated through

(18) $$\begin{array}{c} \psi =\frac{1}{M}\sum_{i=1}^{M}\Gamma_{i}. \end{array}$$

According to (18), *ψ* is the average image, *M* is the number of images, and Γ_*i*_ is the image vector. The corresponding components are maintained with the eigenvalues. These components define the tumor space. The eigenspace is constructed by dragging the image into the tumor space, which results in the formation of the components. As a result, weight vectors are computed. The image dimensions are resized to conform to the standards, and the image is enhanced during the preprocessing stage. The image weight vector is then compared to the tumor weight vector in the image database. The average tumor size is determined and then subtracted from each image in the training set. The result of the subtraction operation is used to form a matrix. The difference between each image and the average image is computed using the formula

(19) $$\begin{array}{c} \phi_{i}=\Gamma_{i}-\Psi ,\;i=1.2.\ldots ,M. \end{array}$$

In (19), the difference between the image and the average image is *ϕ*. The matrix acquired by subtraction is identical to matrix A; it is multiplied by its transpose, and lastly, the covariance matrix C is generated, whose relationship is expressed by

(20) $$\begin{array}{c} C=A^{T}A. \end{array}$$

According to (20), *A* is formed by the disparity between the vectors; for instance, *A*=[*ϕ*_1_, *ϕ*_2_, *ϕ*_3_,…, *ϕ*_*M*_]. Dimensions of *C* matrix are *N* × *N*. The number of image samples, *M,* is used to form matrix *C*. In practice, matrix *C* is the same as *N* × *M*. Alternatively, when the *A* order is equal to *M*, only *M* of *N* is the number of eigenvectors equal to the nonzero value. A covariance matrix is then calculated based on the specific values. Several different images are used to segment the components, and the number of eigenvector classes is subtracted. The number of eigenvector classes measures tumors in the image. To make the feature smaller, the eigenvectors are multiplied by matrix A. As the eigenvectors get smaller, the covariance matrix changes less. However, other features remain the same. Eigenvectors are determined by the accuracy of the images in the image database. Components are groups of eigenvectors. After the components have been obtained, the images in the image database are gathered into the component space, and the image weights are in the same projected image space. An eigencoefficient of a database image is used to determine an image. This is then used to create the component. Calculate the Euclidean distance between the image component and the components collected in the previous step. The tumor is identified as an object whose Euclidean distance is less than the threshold value at the component database. In the case of all Euclidean distances exceeding the threshold, the tumor in the image will not be detected, and the image will be discounted. The convolutional neural network is used as a deep learning technique to improve the results of fragmentation and extraction of the most optimal features in the classification phase. Intending to apply this network, it is necessary to determine the twists and turns, which have three general methods: threshold coefficients wave, adaptive filters, and threshold action potentials scope. This research approach for edge-based segmentation is to use the threshold of the scope of action potentials. The value of this threshold is determined as

(21) $$\begin{array}{c} \left\{\sigma_{n}=\text{median}\left\{\frac{\left|x\right|}{0.6745}\text{Threshold}=3.5\sigma_{n}.\right.\right. \end{array}$$

In equation (21), *x* is the signal recorded by the microelectrode (raw signal) and *σ*_*n*_ is an estimated standard noise deviation. It is important to note that a larger value will be obtained for the threshold by using the standard signal deviation. As a result, some rotations will be mistakenly removed. After selecting the threshold value, the rotations are aligned based on their maximum values. Precise alignment of torsions is a very important and decisive factor in edge-based segmentation with a twist. This neural network needs to be trained. The purpose of this training is to find mapping such as *f* : *R*^*n*^⟶*R* as

(22) $$\begin{array}{c} f\left(v\right)=\sum_{i}^{n}w_{i}\varphi \left(\left|\left|v-C_{i}\right|\right|\right). \end{array}$$

According to (22), *v* ∈ *R*^*n*^ is a 32-point vector for input, and the Gaussian *φ*(0) base function is defined as

(23) $$\begin{array}{c} \varphi \left(v\right)=\exp\left(\frac{-v^{2}}{2\sigma^{2}}\right). \end{array}$$

Then, for each instruction sample's random initial weight values, the corresponding error with each instance is calculated from the gradient descent as

(24) $$\begin{array}{c} e_{i}=t_{i}-y_{i}=t_{i}\sum_{j-1}^{N}w_{j}\varphi \left(\left|\left|v_{i}-C_{j}\right|\right|\right). \end{array}$$

Therefore, the total error of the network for each training input vector *P* of the visual data is equal to *E*=1/2∑_*i*=1_^*p*^|*e*_*i*_|^2^. If the *E* error reaches a lower threshold error, the training ends. This value is determined manually at the beginning. Otherwise, the weights are updated using gradient descent. After completing the training phase with the convolutional neural network, the results are assigned to the class belonging to it.

It should be noted that the main model of this neural network is listed for classification in Section 4. However, it is necessary to determine its layered structure, which includes the input layer (neurons), the hidden layer in which the training operation takes place, and consists of the three inner layers of pooling layers, fully connected layers, and convolve layers. The final test operation is also in the output layer. The difficulty of detecting tumor presence or absence creates a challenge and a search space. In an optimization problem such as tumor detection, the presence or absence of the tumor from the images will be with the *N*_var_ dimension one array *X*_*n*var_, which indicates the current position for its convolve layer in the convolutional neural network. This arrangement is defined as

(25) $$\begin{array}{c} \text{Convolve}=\left[x_{1},x_{2},\ldots ,x_{N\mathrm{var}}\right]. \end{array}$$

The degree of suitability (or profit value) in the current torsion layer or Convolve is obtained by evaluating the function of the tumor (*f*_*p*_) in Convolve. So there are equations (26) and (27).

(26) $$\begin{array}{c} \text{profit}=f_{p}\left(\text{Convolve}\right)=f_{p}\left(x_{1},x_{2},\ldots ,x_{N\mathrm{var}}\right), \end{array}$$

(27) $$\begin{array}{c} j\left(x^{\left(i\right)},\ldots ,x^{\left(n\right)},\theta^{\left(1\right)},\ldots ,\theta^{\left(n\right)}\right)=\frac{1}{2}\sum_{\left(i,j\right):r\left(i,j\right)=1}{\left({\left(\theta^{\left(j\right)}\right)}^{T}x^{\left(i\right)}-y^{\left(i,j\right)}\right)}^{2}+\frac{\lambda }{2}\sum_{i=1}^{n_{m}}\sum_{k=1}^{n}{\left(x_{k}^{\left(i\right)}\right)}^{2}+\frac{\lambda }{2}\sum_{i=1}^{n_{u}}\sum_{k=1}^{n}{\left(\theta_{k}^{\left(j\right)}\right)}^{2}. \end{array}$$

The function of the overall goal in diagnosing tumors or not is in the form of equation (27). In general, we should minimize this function as much as possible. It is a case of removing additional sections to accurately identify the area, which is minimized from

(28) $$\begin{array}{c} j\left(x^{\left(1\right)},\ldots ,x^{\left(n\right)},\theta^{\left(1\right)},\ldots ,\theta^{\left(n\right)}\right). \end{array}$$

As evidence, the structure of the deep convolutional neural network used is an algorithm that maximizes the function of detecting a tumor or not. Using the deep neural network convolution to solve minimization problems such as tumor diagnosis only needs to multiply a minus by the cost function. To start the convolution optimization algorithm, a convolve matrix is generated to the size *N*_pop_*∗N*_var_. Then, a random number of pooling layers is assigned for each of these convolutions. Basically, the pooling layers are between 2 and 5 items. These numbers are used as the upper and lower limits of the pooling allocation to each twisting section in the deep training in different iterations. Another deep convolutional neural network pattern is that they have connected layers at a certain range. Hence, the maximum amplitude of the connected layers in the neural network is called Max_ConnectedLayer_. In an optimization problem, the variable higher limit var_high_ and the lower limit var_low_ will each have a deep layer of Max_ConnectedLayer_, which corresponds to the total layers number, current layers number, and also high and low variables. Therefore, Max_ConnectedLayer_ is defined as

(29) $$\begin{array}{c} {\text{Max}}_{\text{Connected Layer}}=\alpha \times \frac{\text{number of current layers}}{\text{total number of layers}}\times \left({\text{Var}}_{\text{high}}-{\text{Var}}_{\text{low}}\right). \end{array}$$

In (29), *α* is the variable with which the maximum value of Max_ConnectedLayer_ is set. In (28), *θ* is the layers, and in equation (27), *λ* is the evaluator value. Each torsional section in the deep neural network of the convolution traverses only l% of the total detected areas toward the current ideal target and has *φ* radian deviation. These two parameters help each torsion section in the deep convolutional neural network to explore more of the space.*λ* is a random number between 0 and 1, and *φ* is a number between *π*/6 and −*π*/6. When all the torsion sections in the deep convolutional neural network migrate to the target point and new habitat points are identified, each torsion section in the deep convolutional neural network acquires a number of layers of communication. Depending on the number of layers in each torsion section in the deep neural network, a Max_ConnectedLayer_ is determined for it. Now it is necessary to fuzzy this deep neural network of convolution. It is assumed that the image data set is in the form of *M*, which is indicated the number of training images, the average *F*_*i*_ of the images, and the *L*_*i*_ of each image of the *T*_*i*_ vector. Initially, *M* has a number of images, each containing the *M* × *N* dimension. Each image can be displayed in N-dimensional space as *A*=*N∗N∗M*. Therefore, it is necessary to provide a combination layer for the fuzzy part with a deep convolutional neural network, which will be in the form of

(30) $$\begin{array}{c} x_{i}^{\left(l+1\right)}={\left(w_{d}\right)}_{i}^{\left(l+1\right)}{\left(y_{d}\right)}^{\left(l\right)}+{\left(w_{f}\right)}_{i}^{\left(l+1\right)}{\left(y_{f}\right)}^{l}+b_{i}^{\left(l+1\right)}. \end{array}$$

In (30), *y*_*d*_ is the output of the deep convolutional neural network section and *y*_*f*_ is the output of the fuzzy section, which has two sections for weight, *w*_*d*_ and *w*_*f*_. *w*_*d*_ determines the weight of the deep convolutional neural network and *w*_*f*_ determines the weight of the fuzzy part. This layer is used as $\hat{y}$ to combine the deep neural network of the convolution and the fuzzy part, which has a transition function that is considered to be the hyperbolic tangent. In fact, the classification and diagnosis section was presented using an innovative method called fuzzy deep convolutional neural network or FDCNet. What separates the proposed approach from the structure of ANFIS (Neurofuzzy) networks is that, in ANFIS (Neurofuzzy) network structures, the definition of membership functions and fuzzy numbers is based on inputs and some attributes (especially in images where the attributes are considered) and is connected to an extension system that is trained based on a weighted training layer and bias with a transfer or stimulus function. But in the proposed approach structure that performs the detection and classification operation, the fuzzy structure is located on the training layers or hidden layers of the deep neural network section of the convolution after the torsion layer. The filter applied on the torsion layer, the fuzzy layer, the pooling layer, and the completely connected layer is in the form of a 3 × 3 window.

In fact, in this training operation, the interfuzzy coefficients are applied with the layers of convolution to wit the torsion layer, and the output layer displays the classification based on image features as a vector matrix. Definitive processing is performed on the input image to prove the fuzzy uncertainty problem. Also, fragmentation and features extraction based on morphological processing and fractal model uses a three-dimensional matrix in the form of ground truth to accurately and carefully identify features, then reduce the dimensions, and select and extract the features.

### 3.5. The Proposed Approach Diagrams

**Figure 1:**
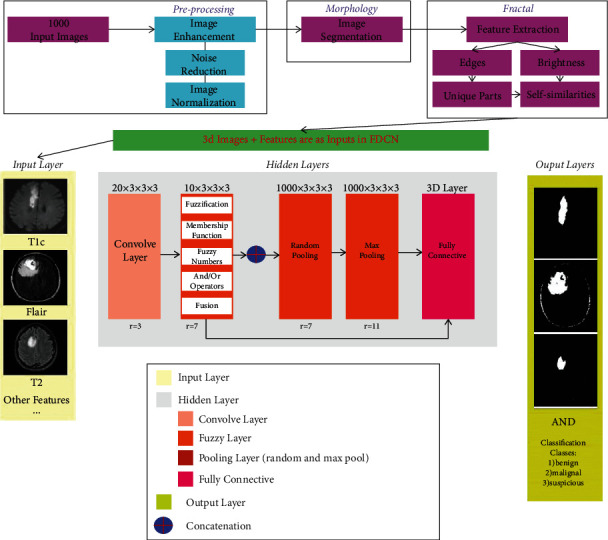
The proposed approach flowchart.

The proposed approach presented in this study has a general trend in Figure 1. This diagram shows that the preprocessing operation starts with 1000 three-dimensional input images, which are from the BraTS data collection and are in the form of DICOM images. Then, the images are improved with the middle filter method to reduce noise and unify their size. Then, the image fragmentation operation based on the intensity of light and edges occurs, which is morphology-based; according to this fragmentation, durable and self-similarity operators of the fractal method begin to work. They can reduce the dimensions, choose the best features, and perform features extraction with high accuracy. The training and testing operation then begins with a deep fuzzy convolutional neural network or a method called FDCNet. This neural network is different from neural-fuzzy networks (ANFIS) in structure because its fuzzy part is considered in the hidden layer in the training section. In contrast, the fuzzy part is first executed in the neural-fuzzy network. Training operations are performed based on input, membership functions, fuzzy numbers, and the rules defined in it.

The FDCNet method inputs, in which the neurons are located, are the three-dimensional images of the data and their features. The training operation is supposed to be performed on three-dimensional images to determine tumors, but in the final classification, in addition to the original image, features are needed to create benign, malignant, and suspicious classes. The FDCNet structure, after reading the data from the input layer, is to create a hidden layer including 20-channel twist layers and a 3 × 3 filter, which is 3 × 3 × 3 in three-dimensional images and has an Astros rate of 3 (*r* = 3). Next is the fuzzy layer, along which fuzzy data training in the twisted layer determines membership functions, fuzzy numbers, And/Or rules, and then fusion. Also, its Astros rate is 7 (*r* = 7), and the filter applied to it is 3 × 3.3. There are also 10 layers in this fuzzy section. Then the training and classification operation in the pooling layer is applied in two parts: random pooling and max pooling, which has 1000 data equal to the layers for training (i.e., 100% of the data are trained, and any image from the data set can be tested on). The filter applied to it is 3 × 3 with an Astros rate of 11. Then there is the 3-dimensional layer of the data test as a completely connected layer, which is also affected by the fuzzy layer. The data tests performed in this layer transfer to the output layer and display the tumor area visually and then create classes to display the type of tumor as malignant, benign, and suspicious.

## 4. Results and Discussion

The set of brain tumor data used in this study is BraTS data. It has 145 files for individuals undergoing various MRI imaging procedures. This data collection has four versions ranging from 2012 to 2018, with each version increasing the quantity of data pieces and their quality. The primary data is in DICOM format, converted to JPEG for better usage with the DICOM Viewer program in this study. Three-dimensional graphics are used as input. Due to the vast image size in the BraTS data set, this study employs 1000 video input samples to validate the suggested technique. Several images have been used that will be displayed in the results section. The simulation takes place in MATLAB 2015b and will run on a system with a 7-core processor with 6 Mbps cache and 3.6 MHz and 6 GB of memory in Windows 8. First of all, the structure of the deep neural network of fuzzy convolution should be considered, and the number of layers and the number of neurons should be determined. Initially, 32 primary neurons are considered in the input layer, which includes all the features of BraTS data. There are settings in the hidden layer with three main sections in the neural network, deep fuzzy convolution depth, namely, twisting, pooling, and completely connected. The sum of these layers is 4 cases creating a 3 × 3 torsional matrix. This means that, in each of the 4 layers, there is also a 3 × 3 matrix. The torsion layer is a single layer, and the pooling layer consists of two layers, one part of which is considered maximal or the so-called *maxpool*, and the other part is random pooling that can teach each of the features at random. So there is one torsion layer, two pooling layers, and one fully connected layer, and the output layer contains any feature based on distinguishing features from MRI images. These layers are also connected to a fuzzy structure. Training section layers are arranged in the following order: a twisting layer, a deep fuzzy layer with membership functions that are calculated from the output features, the original three-dimensional images, two layers of pooling layer including the random part and the maxpool section, and the fully connected layer. There is a problem called centroid, which is considered in the principles of classification and even clustering to perform detection and work tracking. The structure is individual, that is, 3 × 3, 5 × 5, and 7 × 7. A house or pixel is placed in the middle, the adjacent houses are analyzed, and that central pixel is considered the centroid. It is filtering on the layers of the training or hidden section measured as 3 × 3 in small dimensions. The results and simulation outputs performed will be placed in this section. First, all images will be read. The operation begins in the training and testing phase with the neural convolutional network and features extraction with the fractal model.

**Figure 2:**
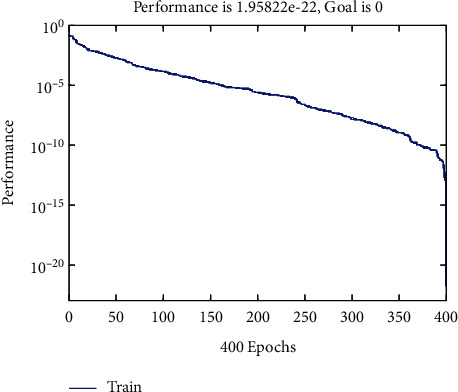
Performance diagram to achieve convergence and end goal in the training phase.

**Figure 3:**
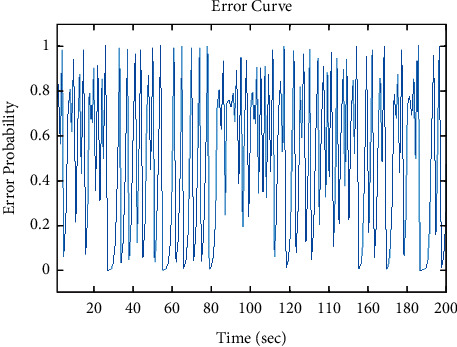
The error graph at the time of training from the whole data set is based on an error probability at the simulation time.

**Figure 4:**
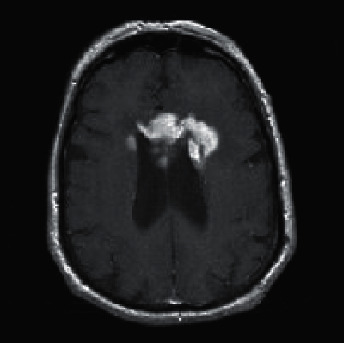
Input image.

**Figure 5:**
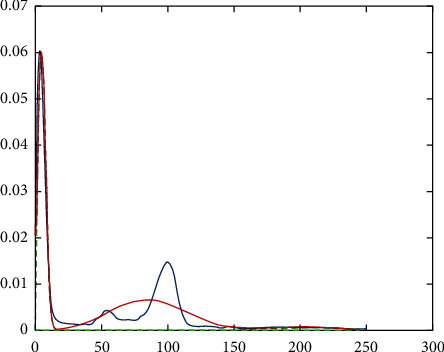
Histogram and color channel separation of the input image.

**Figure 6:**
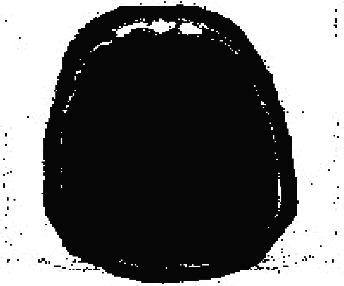
Converted image from RGB to gray surface.

It should be noted that the three-dimensional MRI image will be processed. Then for the final classification, the features of the fragmentation are used, which are a combination based on morphological processing and a fractal model that creates a ground truth three-dimensional matrix.  Figure 2 shows the performance chart to achieve convergence and the ultimate goal in the training phase. To test the data, an input image needs to be considered. 70% of the data are trained, and 30% are tested. But one of the images needs to be shown visually (see Figure 3). We used one of the images in the data set, shown in Figure 4. Then, the histogram and the separation of the color channels of that image are calculated, which is similar to Figure 5. This histogram and color channel separation show the red, green, and blue color channels based on 256 colors on a threshold. The image is then normalized and converted from color mode or RGB color space to gray, resulting in Figure 6.

**Figure 7:**
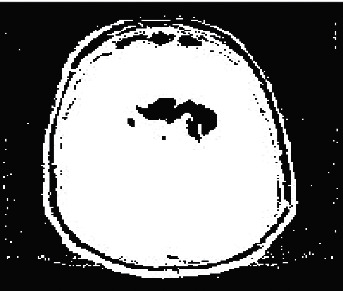
The result of image fragmentation based on the fractal model.

**Figure 8:**
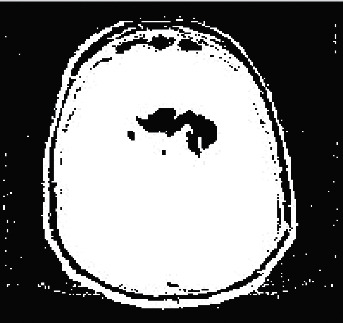
The result of final fragmentation in the neural network training phases of deep fuzzy convolution.

**Figure 9:**
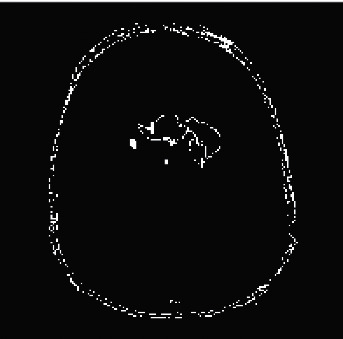
The final result of the proposed method.

**Figure 10:**
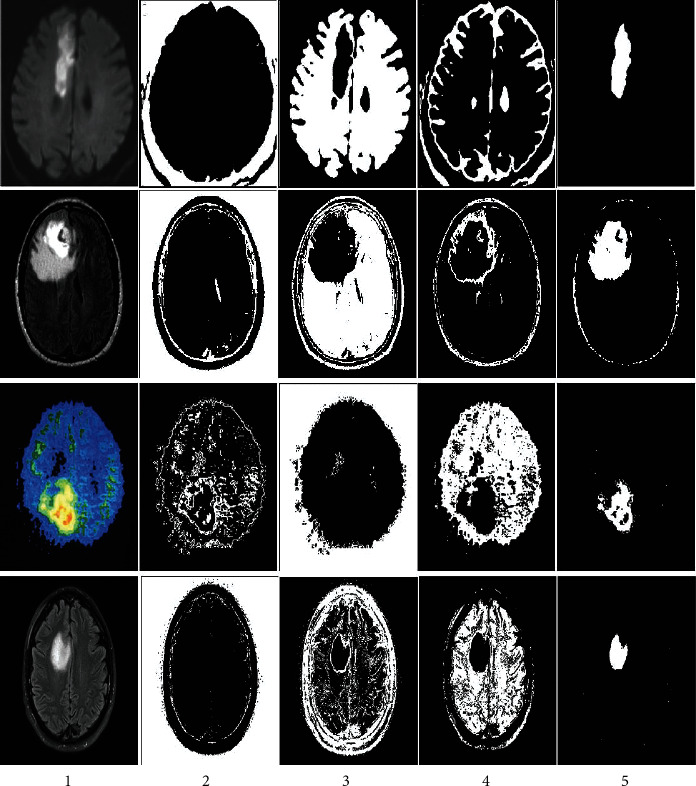
The results of the proposed method on other images.

Then segmentation based on the fractional model by local features, including texture, light intensity, and image edge, based on two factors including self-similarity and durability, is as shown in Figure 7. The fragmentation is performed by identifying three features, including light intensity, edges separated from the original image, and texture, which is shown in Figure 8. The used features are based on edge finding (connected points in another object), light intensity, and texture. Three local features are used for fragmentation. In the basic sciences, in comparison to the methods that use more number of features, those methods with lower feature numbers with appropriate answers have higher scientific validity. Finally, the fuzzy deep neural network will be used for the final diagnosis, aiming to improve the segmentation and select the optimal class of features to detect the features in the MRI images in the image, resulting in Figure 9. As shown in Figure 9, the image segmentation and classification operations are well performed, and the area of tumor features is identified. In Figure 10, the method presented is applied to several other images in the data set.

**Figure 11:**
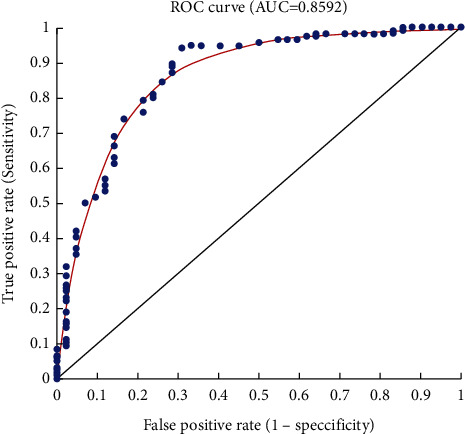
ROC diagram and AUC rate.

In addition to the features mentioned above, it is essential to consider local characteristics, including light intensity, edges, and any other characteristic that may have been identified during the segmentation and feature extraction steps, as well as tumor classification operations, such as benign, malignant, and suspicious. This is where the three-dimensional ground truth matrix begins to work using the image properties to consider the classification. This curve measures the system's ability to classify or cluster data. It is a graphical depiction of the degree of sensitivity in a binary classification system with variable separation thresholds or the ratio of true to erroneous predictions. In addition to graphing true positive predictions versus incorrect positive predictions, the receiver operating characteristic (ROC) curve can also be seen. An area under the receiver operating characteristic curve is a numerical value used to quantify and analyze some aspect of performance. Its value varies between 0 and 1. AUC stands for the area under the curve. A value of 0.5 indicates no prediction, whereas values between 0.7 and 1 indicate good predictions, classifications, or groupings. In Figure 11, the AUC value is less than one, suggesting that the suggested technique has been optimized as much as possible. A combination of pieces resembling malignant tumors in the available data and the approach described in this research produced a series of small inaccuracies that are detectable when the fitting line is not matched (Table 1). These blue circles represent the data's qualities, while the red lines represent the data's fit to the ROC diagram. When the data is a little out of reach, errors occur. The middle line also includes regression. The area between the ROC peak and the regression line is the AUC.  Table 2 shows the comparison of the proposed method with several other new methods that were studied in previous studies. This comparison is based on the accuracy criterion in terms of percentage. All methods use the same data set, the BraTS.

## 5. Conclusion

Smartification principles in the construction of intelligent medical diagnostic systems to have reliable and rapid procedure have been required. Intelligent medical diagnostic systems could reduce human error and assist physicians in diagnosing. This identification and early detection lead to health recognition and further care until complete recovery. Cancer masses that form in different body parts do not have a regular shape or specific principles. Imaging other body areas can help identify the size and area of these tumors. It is also possible to estimate malignant and benign conditions using medical courses. These tumor masses, which are one of the world's leading death causes, need to be diagnosed as accurately as possible. Therefore, building intelligent systems is vital and inevitable. In this study, an attempt was made to use brain imaging to produce MRI images that can contain tumors. This study's data set includes BraTS, a series of standard MRI images of the brain surface that have the same features in light intensity, brightness, and color states. Using the principles of image processing and machine learning, a comprehensive system for detecting brain tumors from the level of MRI images can be provided. The proposed approach method initially provided a preprocessing step to reduce noise and smooth image size. In the following, the fragmentation operation based on morphology and, at the same time, the feature extraction operation with the fractal model are presented. In the continuation of the diagnostic and classification operations, the innovative deep neural network of fuzzy convolution or FDCNet has been considered. Its accuracy results have been 98.68.

This method offered a functional advantage over previous methods, including tremendously generative adversarial networks (GAN), progressively growing adversarial networks (PGGAN), convolutional deep neural networks with extensive data enhancement, using super-pixel images with fuzzy C-Means methods, and convolutional neural networks, as well as a discrete wavelet transform method based on convolutional neural networks (DWT-CNN). The existence of segmentation that is comparable to malignant masses in the available data, together with the approach used in this study, has resulted in a series of small inaccuracies that can be seen on the fitting line. An error happens in some regions when the data is out of reach. Based on the results, the presented method's accuracy, sensitivity, and AUC are 98.69%, 94.88%, and 85.92%, respectively. For the future of the study, the presented method should be extended with edge detection methods. Moreover, the optimization for the clustering method can be done using metaheuristic search algorithms such as Genetic Algorithm and particle swarm optimization [55].

## Figures and Tables

**Table 1:** Evaluation results.

Parameter	PR
Accuracy	98.6891%
Sensitivity	94.8766
Mean square error	0.8598
Peak signal-to-noise ratio	21,2646 dB
Signal-to-noise ratio	26,8642 dB
Area under curve	0.8592

**Table 2:** The results of the proposed method with other methods.

Reference	Proposed method	Precision (%)
Chen et al., 2019 [3]	Using generative adversarial networks (GAN)	94.5
Han et al., 2019–2020 [4]	PGGAN	95
Özyurt et al., 2019 [5]	Convolutional neural network with a combinational method called NS-CNN-EMFSE	96.52
Sajjad et al., 2019 [9]	Deep convolutional neural network with expanded data amplification	95.5
Özyurt et al., 2020 [13]	Using a super-pixel image with C-means fuzzy combinational method and convolutional neural network	98.33
Amin et al., 2020 [54]	Discrete wavelet transform method based on convolutional neural network (DWT-CNN)	98
Ahmadi et al., 2020 [14]	Optimization of quantum matched-filter techniques and deep spiking neural Networks (QAIS-DSNN methods)	91.92
The proposed method	The fractal segmentation method is based on a deep fuzzy convolutional neural network or FDCNet	98.68

## Data Availability

Data are available and can be provided over the emails querying directly to the corresponding author (y pourasad@uut ac ir).
